# Hydrocortisone combined with fludrocortisone for treatment of adults with septic shock: an updated meta-analysis and systematic review

**DOI:** 10.3389/fmed.2026.1755626

**Published:** 2026-02-11

**Authors:** Alin Sun, Xiang Gao, Zhipeng Gao, Qinghai Zhang, Wenzheng He

**Affiliations:** 1 Department of Critical Care Medicine, Weifang People’s Hospital, Weifang, Shandong, China; 2 Department of Critical Care Medicine, The First Affiliated Hospital of Shandong Second Medical University, Weifang, Shandong, China

**Keywords:** fludrocortisone, hydrocortisone, meta-analysis, mortality, septic shock

## Abstract

**Purpose:**

To evaluate the efficacy and safety of hydrocortisone combined with fludrocortisone in the treatment of septic shock in adults.

**Methods:**

We searched PubMed, Embase, Web of Science, and the Cochrane Library for studies on hydrocortisone combined with fludrocortisone in the treatment of septic shock in adults. Two investigators independently screened studies, extracted data, and assessed the risk of bias of the included studies. A meta-analysis was performed using RevMan 5.3 and STATA 17.0 software.

**Results:**

A total of eight studies (5 RCTs and 3 N-RCTs) were included. Stratified analysis by study design and comparator type revealed that in the HC + FC vs. Placebo subgroup (derived solely from RCTs), the combination significantly reduced 28-day mortality [RR 0.84; 95% CI (0.76, 0.94); *p* = 0.002], 90-day mortality [RR 0.82; 95% CI (0.71, 0.94); *p* = 0.006], and in-hospital mortality [RR 0.85; 95% CI (0.77, 0.94); *p* = 0.002]. In contrast, for the HC + FC vs. HC alone subgroup (addressing incremental benefit), no significant survival advantage was observed in either RCTs (*n* = 553) or N-RCTs (*n* = 88,666, 28-day mortality RR 0.99, *p* = 0.79). Regarding safety, HC + FC was associated with a higher reinfection rate compared to placebo (RR 1.13, *p* = 0.03) but not when compared to HC alone (*p* = 0.19). No significant increase in gastrointestinal bleeding or reduction in ICU/hospital length of stay was identified across all tiers of evidence.

**Conclusion:**

Evidence primarily from RCTs indicates that HC + FC is associated with improved survival compared to placebo in septic shock. However, large-scale observational data suggest no significant incremental benefit over hydrocortisone alone. While the combination appears safe regarding gastrointestinal bleeding, the increased reinfection risk compared to placebo warrants caution. Given the non-causal nature of observational findings, these results are suggestive rather than definitive. Future head-to-head trials are essential to confirm the marginal efficacy of fludrocortisone supplementation.

**Systematic review registration:**

https://www.crd.york.ac.uk/PROSPERO/view/CRD420251001999, Identifier: CRD420251001999

## Introduction

Sepsis is a leading cause of death in intensive care unit (ICU) patients, with high morbidity and mortality rates worldwide (1). Previous studies (2–4) have shown that although anti-infective treatment and organ support technologies have made great progress, the mortality rate of sepsis is still as high as 30 to 70%. In addition, 50% of septic shock patients may experience long-term cognitive impairment even after hospital discharge (5, 6). Moreover, the treatment of sepsis is also costly and consumes substantial healthcare resources, which seriously affects the quality of life of patients. This brings a heavy economic and psychological burden to patients’ families and society.

Experimental and clinical evidence has suggested that sepsis is associated with dysregulation of the hypothalamic-pituitary-adrenal axis (7). Glucocorticoids can regulate the immunologic response through anti-inflammatory effects (8) and improve vascular responsiveness to catecholamines (9). Due to these potential benefits, corticosteroids have been used to treat patients with severe infections. The 2021 Surviving Sepsis Campaign Guidelines (10) recommend the use of corticosteroids for adults with septic shock and ongoing requirement for vasopressor therapy. However, the strength of the recommendation is weak, and the quality of evidence is moderate. Although many relevant studies (11–14) have been conducted, whether glucocorticoids can improve outcomes or prevent progression to septic shock in seriously ill patients remains inconclusive to date.

Recent studies (15, 16) have shown that hydrocortisone combined with fludrocortisone can effectively reduce mortality in patients with sepsis. This combination may provide clinical benefits in the treatment of septic shock. However, some studies (17, 18) have reported contradictory findings. Therefore, we conducted a meta-analysis to evaluate the efficacy and safety of hydrocortisone combined with fludrocortisone in the treatment of septic shock, to provide stronger evidence for clinical practice.

## Methods

### Protocol and registration

This systematic review is reported according to the PRISMA 2020 statement: an updated guideline for reporting systematic reviews (19). Our study protocol was pre-registered with PROSPERO (CRD420251001999). The pre-specified primary outcome was 28-day mortality, 90-day mortality, and in-hospital mortality. Secondary outcomes included incidence of reinfection, gastrointestinal bleeding, and length of ICU and hospital stay. Furthermore, we pre-planned subgroup analyses based on study design (RCT vs. N-RCT) and comparator type (HC + FC vs. Placebo vs. HC monotherapy) to explore the incremental benefit of fludrocortisone.

### Eligibility criteria

Studies were included if they met the following criteria: (1) population: adult patients with septic shock; (2) intervention: hydrocortisone-plus-fludrocortisone therapy; (3) comparison: placebo or hydrocortisone monotherapy; (4) design: observational studies, retrospective studies, or randomized trials; (5) outcomes: the primary outcomes were mortality (including hospital mortality, 28-day mortality, and ICU mortality). Secondary outcomes included length of ICU stay, length of hospital stay, and adverse events (including secondary infection and gastrointestinal bleeding).

### Information sources

We searched PubMed, Embase, Web of Science, and the Cochrane Library from inception to March 1, 2025. We used a combination of medical subject headings (MeSH) and keyword terms to search. The full search strategy for PubMed is provided in Supplementary Table 1 and was tailored for other databases. In addition, we conducted a manual search of reference lists of eligible studies and previous reviews.

### Study selection

We removed duplicate records from the initial search, screened titles and abstracts for relevance, and labeled records as included, excluded, or uncertain. We reviewed the full texts labeled included or uncertain to identify eligibility of studies. This process was independently carried out by two researchers. In case of disagreement during this process, a third investigator was consulted to reach consensus.

### Data extraction

Data extraction was performed using a standardized Excel spreadsheet (Microsoft Corporation) and confirmed by a second reviewer. The relevant information extracted from each study was as follows: author, year, study design, clinical setting, study population, number of patients, hydrocortisone regimen, time of hydrocortisone initiation, and outcomes. Any discrepancies were resolved by discussion between the two researchers. If the two researchers were unable to resolve the discrepancy, then the third researcher resolved it by reviewing the original study.

### Quality assessment

The quality assessment of the included studies was conducted using the methods recommended in the “Cochrane Handbook for Systematic Reviews” to assess the risk of bias. Any disagreements were resolved by discussing with a third reviewer.

### Statistical analysis

Statistical analyses were performed using RevMan 5.3 (Nordic Cochrane Centre) and STATA 17.0 (StataCorp LLC). For dichotomous outcomes, we calculated the pooled risk ratio (RR). For continuous outcomes, the mean difference (MD) was utilized. For continuous data reported as medians and interquartile ranges (IQRs), we estimated the means and standard deviations (SDs) using the validated mathematical framework provided by Wan et al. (44). Skewness was assessed by evaluating the position of the median within the IQR; in cases of suspected non-normal distribution, we maintained a conservative approach by employing a random-effects model to account for the additional uncertainty introduced by the transformation. All results are presented with corresponding 95% confidence intervals (CIs). To address potential clinical and methodological heterogeneity, a random-effects model was systematically employed for all pooled analyses. Statistical heterogeneity was assessed using the *I*^2^ statistic and the *χ*^2^ test, with *I*^2^ > 50% indicating substantial heterogeneity. All analyses were stratified by study design [randomized controlled trials (RCTs) vs. non-randomized controlled trials (N-RCTs)] to prevent the pooling of disparate levels of evidence. Publication bias was evaluated using funnel plots and Egger’s linear regression test for outcomes. Sensitivity analyses were conducted using the “leave-one-out” method to ensure the robustness of the findings and to evaluate the influence of individual large-scale cohorts.

## Results

### Study selection and study characteristics

**Figure 1:**
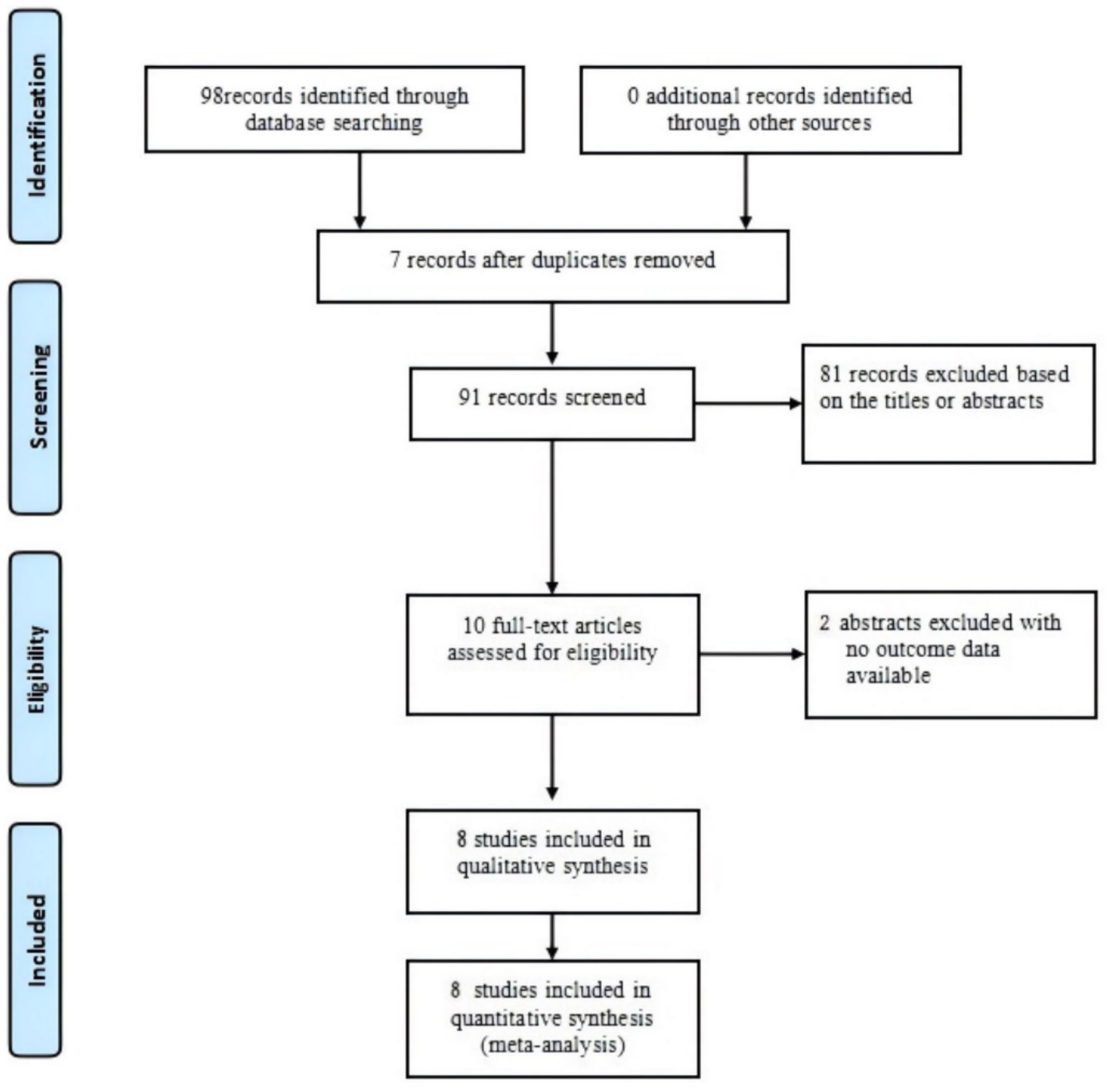
Study inclusion flowchart.

**Figure 2:**
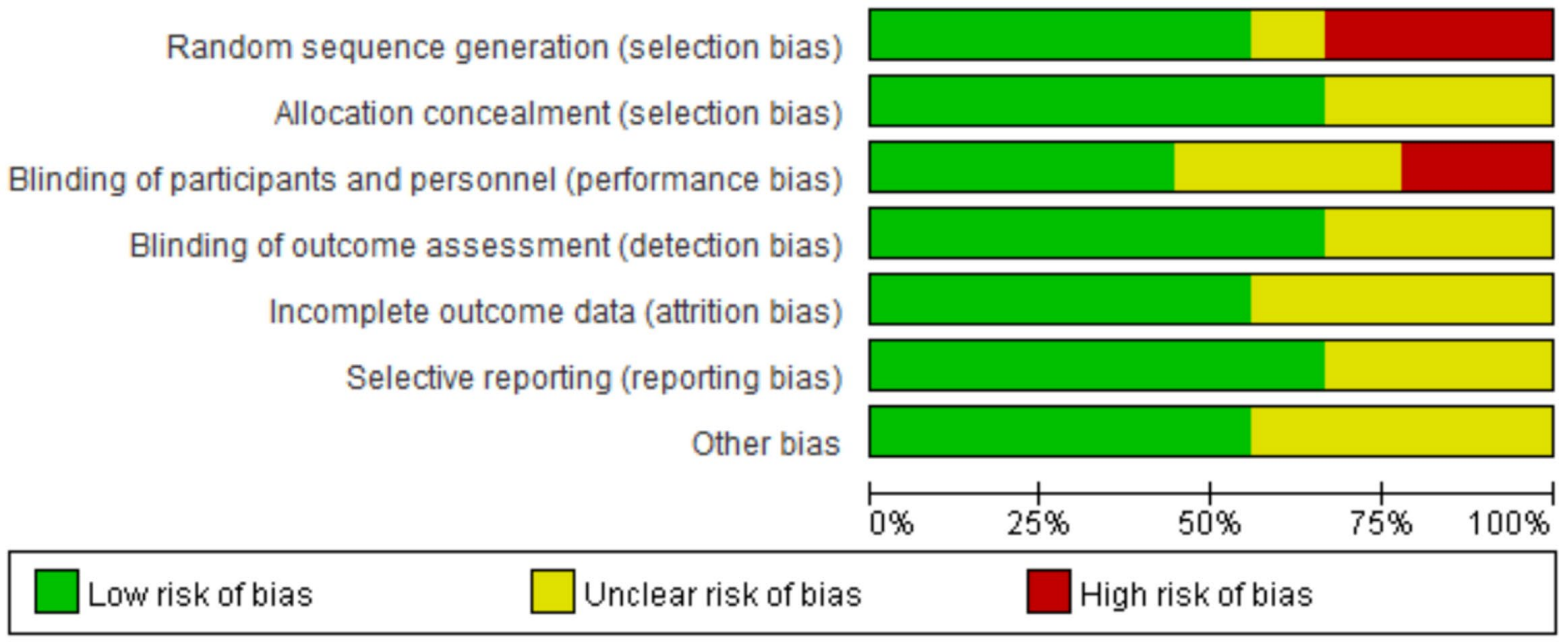
Quality evaluation of incorporated literature.

The study selection process is presented in Figure 1. The initial search yielded 98 records. After removing duplicates, 91 records were retained. By screening the titles and abstracts, we excluded 82 records for not meeting the inclusion criteria. The full texts of the remaining nine articles were examined for eligibility. Finally, eight studies were included in the analysis (15, 16, 18, 20–24). Characteristics of the included studies are summarized in Table 1. Details of the quality assessment of included studies are presented in Figure 2.

**Table 1:** Baseline characteristics of studies included in the meta-analysis.

Study	Country	Type of study	Control intervention	Sample size	Patient characteristics (T: trial/C: control)			Timing of treatment initiation	Outcome indicators
				(T: trial/C: control)	Age (year)	Sex (M: men/W: women)	Severity scoring system		
Annane et al. (20)^a^	France	RCT	Placebo	T: 150 C: 149	T: 62 ± 15 C: 60 ± 17	T: (M: 96/W: 54) C: (M: 104/W: 45)	SAPS II T: 60 ± 19 C: 57 ± 19	Within 3 to 8 h after the occurrence of shock	28-day mortality rate; hospital mortality rate; reinfection; gastrointestinal bleeding
Annane et al. (21)^a^	France	RCT	Applying hydrocortisone alone	T: 245 C: 264	T: 64.0 ± 2.7 C: 63.9 ± 2.7	T: (M: 167/W: 78) C: (M: 163/W: 101)	SAPS T: 58.9 ± 3.2 C: 60.4 ± 3.1 SOFA T: 10.6 ± 0.7 C: 10.4 ± 0.7	Within 24 h after meeting the criteria for septic shock, they were randomly assigned and began receiving treatment	Hospitalization mortality rate, re-infection, length of hospital stay, length of stay in ICU
Annane et al. (15)^a^	France	RCT	Placebo	T: 614 C: 627	T: 66 ± 14 C: 66 ± 15	T: (M: 402/W:212) C: (M: 424/W: 203)	SAPS II T: 56 ± 19 C: 56 ± 19 SOFA T: 12 ± 3 C: 11 ± 3	Within 24 h after meeting the criteria for septic shock, they were randomly assigned and began receiving treatment	28-day mortality rate, 90-day mortality rate, hospitalization mortality rate, re-infection, gastrointestinal bleeding
Labib et al. (22)^a^	Egypt	RCT	Applying hydrocortisone alone	T: 22 C: 22	T: 61.8 ± 4.9 C: 60.6 ± 5.8	T: (M: 14/W:8) C: (M: 15/W: 7)	SOFA T: 8.59 ± 1.79 C: 8.36 ± 1.76	Patients who meet the inclusion criteria (i.e., adult patients diagnosed with septic shock) must be randomly assigned and commence treatment within 24 h of diagnosis	Reinfection, gastrointestinal bleeding, length of stay in ICU
Bosch et al. (16)^a^	US	Retrospective	Applying hydrocortisone alone	T: 2,280 C: 85,995	T: 63.6 ± 14.1 C: 66.6 ± 14.1	T: (M: 1,239/W: 1,041) C: (M: 43,859/W: 42,136)	Elixhauser comorbidity score T: 5.6 ± 2.2 C: 5.6 ± 2.2	The study compared whether fludrocortisone was added simultaneously on the same day when hydrocortisone was initiated	28-day mortality rate; hospital mortality rate
John et al. (23)^a^	US	Retrospective	Applying hydrocortisone alone	T: 70 C: 70	T: 64.0 ± 12.1 C: 66.4 ± 15.1	T: (M: 40/W:30) C: (M: 34/W: 36)	SAPS II T: 50.7 ± 16.7 C: 51.3 ± 23.5 SOFA T: 12.3 ± 4.5 C: 10.9 ± 6.8	Hydrocortisone and fludrocortisone must be administered on the same day. The time interval between the two administrations should not exceed 12 h; otherwise, it will not be regarded as combined medication	28-day mortality rate, 90-day mortality rate, in-hospital mortality rate, hospital stay, ICU stay time
Lock et al. (18)^a^	US	Retrospective	Applying hydrocortisone alone	T: 114 C: 137	T: 60.2 ± 14.4 C: 58.6 ± 13.4	T: (M: 56/W:58) C: (M: 82/W: 55)	SAPS II T: 42.8 ± 15.8 C: 44.1 ± 12.7 SOFA T: 9.4 ± 3.7 C: 9.7 ± 2.8	All patients began using hormones after requiring norepinephrine (or other vasopressor drugs) to maintain blood pressure. For the combined group, fludrocortisone (FC) must be initiated within 24 h after the administration of hydrocortisone	28-day mortality rate, 90-day mortality rate, gastrointestinal bleeding, in-hospital mortality rate, time spent in ICU, hospital stay
Heming et al. (24)^a^	France	RCT	Placebo	T: 283 C: 279	T: 64 ± 14 C: 65 ± 15	T: (M: 200/W:83) C: (M: 193/W: 86)	SAPS II T: 56 ± 19 C: 56 ± 18 SOFA T: 12 ± 3 C: 12 ± 3	Participants were enrolled and began receiving treatment within 24 h after meeting the criteria for “possible or confirmed septic shock.” Once enrolled, the patients undergo a 7-day treatment course	28-day mortality rate, 90-day mortality rate, hospitalization mortality rate, re-infection, gastrointestinal bleeding

NA, not acquire; US, The United States of America; RCT, randomized controlled trial; APACHE II, Acute Physiology And Chronic Health Evaluation II; SAPS, Simplified Acute Physiology Score; SOFA, Sequential Organ Failure Assessment; ICU, intensive care unit.

a Hydrocortisone: 50 mg, intravenous injection, once every 6 h; Fludrocortisone: 50 μg, oral administration, once a day.

### Effect evaluation

#### 28-day mortality rate

**Figure 3:**
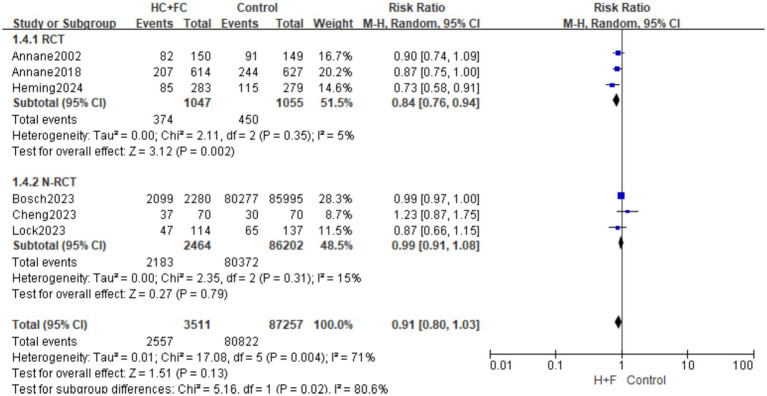
Forest plot of 28-day mortality rate.

A total of six articles (15, 16, 18, 20, 23, 24) reported the 28-day mortality rate and 91,416 patients were included. The primary analysis of three RCTs (*n* = 2,102) demonstrated that the combination of hydrocortisone and fludrocortisone significantly reduced the risk of 28-day mortality [RR: 0.84; 95% CI: (0.76, 0.94); *p* = 0.002]. Heterogeneity within this subgroup was negligible (*I*^2^ = 5%; *p* = 0.35), indicating high consistency among the trial findings (Figure 3).

The secondary analysis comprised three observational studies with a total of 88,666 patients. In this subgroup, the association between HC + FC and 28-day mortality did not reach statistical significance [RR: 0.99; 95% CI: (0.91, 1.08); *p* = 0.79]. The internal heterogeneity was low-to-moderate (*I*^2^ = 15%; *p* = 0.31). Notably, this subgroup was heavily influenced by the large-scale Bosch (2023) cohort, and the wide confidence interval reflects the inherent uncertainty in these observational data (Figure 3).

The overall pooled effect [RR 0.91; 95% CI: (0.80, 1.03); *p* = 0.13] showed considerable heterogeneity (*I*^2^ = 71%; *p* = 0.004). The test for subgroup differences revealed a significant interaction (*I*^2^ = 80.6%; *p* = 0.02). This statistical divergence suggests that the therapeutic efficacy observed in randomized settings is significantly more robust than the effects estimated from large-scale retrospective cohorts (Figure 3).

Visual inspection of the funnel plot and Egger’s test revealed no evidence of significant publication bias (*p* > 0.05; Supplementary Figures S3B,C). “Leave-one-out” sensitivity analysis confirmed the robustness of the findings; the omission of any individual study—including the dominant Bosch (16) cohort—did not significantly alter the pooled RR or the overall conclusion (Supplementary Figure S3D).

#### Hospital mortality rate

**Figure 4:**
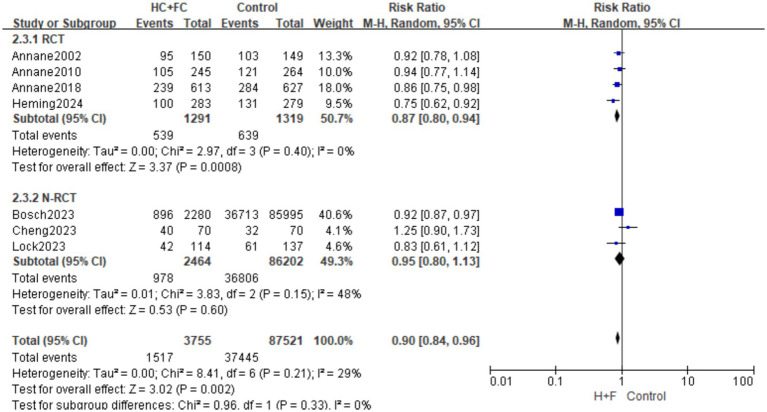
Forest plot of in-hospital mortality rate.

Seven studies (15, 16, 18, 20, 21, 23, 24) reported in-hospital mortality, and a total of 91,924 patients were included in the study. The meta-analysis of four RCTs (*n* = 2,610) demonstrated that the combination of HC + FC significantly reduced the risk of in-hospital mortality compared with the control group [RR: 0.87; 95% CI: (0.80, 0.94); *p* = 0.0008]. No statistical heterogeneity was observed within this subgroup (*I*^2^ = 0%; *p* = 0.40) (Figure 4).

In the subgroup of three observational studies (*n* = 88,666), HC + FC showed a consistent but non-significant trend toward reduced in-hospital mortality [RR: 0.95; 95% CI: (0.80, 1.13); *p* = 0.60]. Moderate heterogeneity was noted among these studies (*I*^2^ = 48%; *p* = 0.15, Figure 4). As with other mortality outcomes, this subgroup was notably influenced by the large-scale Bosch (16) cohort, which contributed 40.6% of the total weight.

The overall pooled effect indicated a significant reduction in the risk of in-hospital death [RR: 0.90; 95% CI: (0.84, 0.96); *p* = 0.002], with low overall heterogeneity (*I*^2^ = 29%; *p* = 0.21). The test for subgroup differences showed no significant interaction between the RCT and N-RCT designs (*p* = 0.33; *I*^2^ = 0%), suggesting that the direction of effect was uniform across both experimental and observational evidence (Figure 4).

Funnel plot symmetry and Egger’s test results (*p* > 0.05; Supplementary Figures S4B,C) indicated no significant publication bias for in-hospital mortality. “Leave-one-out” sensitivity analysis confirmed the robustness of the findings, as the pooled RR remained stable (Supplementary Figure S4D) without being disproportionately influenced by any single study, including the large-scale Bosch (16) cohort.

#### 90-day mortality rate

**Figure 5:**
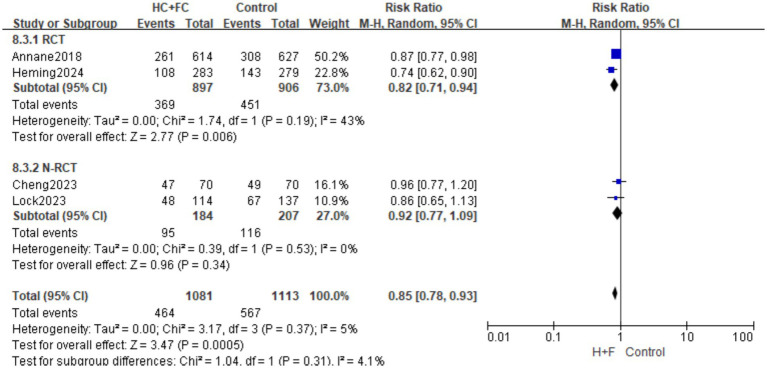
Forest plot of 90-day mortality rate.

Four studies (15, 18, 23, 24) reported 90-day mortality, and involving 1,081 patients. The primary analysis of two RCTs demonstrated that the combination therapy significantly reduced the risk of 90-day mortality [RR: 0.82; 95% CI: (0.71, 0.94); *p* = 0.006, Figure 5]. Moderate heterogeneity was observed within this subgroup (*I*^2^ = 43%; *p* = 0.19), and the RCTs contributed a combined weight of 73.0% to the overall analysis.

The secondary analysis included two observational studies. In this subgroup, the association between HC + FC and 90-day mortality did not reach statistical significance [RR: 0.92; 95% CI: (0.77, 1.09); *p* = 0.34, Figure 5]. No statistical heterogeneity was found among these observational findings (*I*^2^ = 0%; *p* = 0.53).

The overall pooled effect across all four studies showed a significant survival benefit for patients receiving HC + FC [RR: 0.85; 95% CI: (0.78, 0.93); *p* = 0.0005, Figure 5], with very low overall heterogeneity (*I*^2^ = 5%; *p* = 0.37). The test for subgroup differences indicated no significant interaction between the RCT and N-RCT designs (*p* = 0.31; *I*^2^ = 4.1%), suggesting that the direction of the effect remained consistent regardless of the study design.

Visual inspection of the funnel plot and Egger’s test results yielded no significant evidence of publication bias for 90-day mortality (*p* > 0.05; Supplementary Figures S5B,C). “Leave-one-out” sensitivity analysis confirmed the robustness of the findings, with the pooled RR remaining stable between 0.82 and 0.86 upon sequential exclusion of individual studies (Supplementary Figure S5D). These results indicate that the survival benefit at 90 days is reliable and not driven by any single study.

### Safety assessment

#### The incidence rate of re-infection

**Figure 6:**
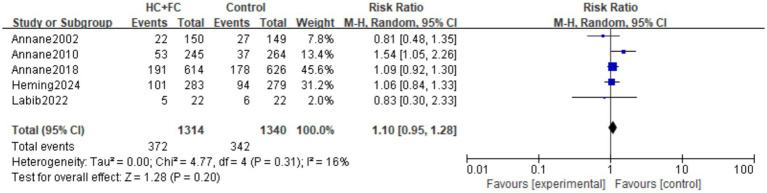
Forest plot of re-infection rate.

Five RCTs (15, 20–22, 24) involving 2,654 patients reported the incidence rate of re-infection. The pooled analysis demonstrated that the combination of hydrocortisone and fludrocortisone did not significantly increase the risk of secondary infections compared with the control group [RR: 1.10; 95% CI: (0.95, 1.28); *p* = 0.20]. Low statistical heterogeneity was observed among the included trials (*I*^2^ = 16%; *p* = 0.31; Figure 6). Due to the fact that different research reports describe different sites of re-infection, we conducted a further site-specific analysis. The results showed that there was no statistically significant difference in the incidence rates of catheter-related infections, pulmonary infections, bloodstream infections, and urinary tract infections between the hydrocortisone combined with fludrocortisone group and the control group (Table 2). However, it should be noted that the clinical definitions of secondary infection varied across the included studies, which should be considered when interpreting these results.

**Table 2:** The results of the meta-analysis of the infection sites.

Infection site	The number of included studies	Heterogeneity test		Statistical results		
		*p*	*I* ^2^	RR	95% CI	*p*
Catheter-related infection	3 (15, 20, 24)	0.98	0%	1.14	0.85, 1.51	0.39
Pulmonary infection	4 (15, 20, 21, 24)	0.79	0%	1.14	1.00, 1.31	0.06
Blood infection	3 (15, 21, 24)	0.73	0%	1.09	0.84, 1.42	0.52
Urinary tract infection	4 (15, 20, 21, 24)	0.27	24%	1.37	0.95, 1.99	0.10

RR, relative risk; 95% CI, 95% confidence interval.

Visual inspection of the funnel plot (Supplementary Figure S6B) and Egger’s linear regression test (Supplementary Figure S6C) revealed no significant evidence of publication bias (*p* > 0.05). Additionally, “leave-one-out” sensitivity analysis (Supplementary Figure S6D) confirmed the robustness of the findings, as the pooled RR remained statistically stable and no single study disproportionately influenced the overall effect size.

#### The incidence rate of gastric and duodenal hemorrhage

**Figure 7:**
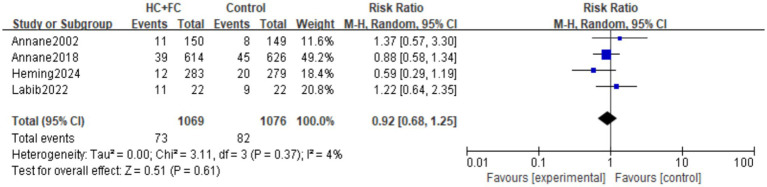
Forest plot of the incidence rate of gastric and duodenal bleeding.

Four RCTs (15, 16, 20, 24) involving 2,145 patients provided data on the incidence of gastric and duodenal hemorrhage. The pooled analysis showed no significant difference in the risk of gastric and duodenal hemorrhage between the HC + FC group and the control group [RR: 0.92; 95% CI: (0.68, 1.25); *p* = 0.61, Figure 7]. Statistical heterogeneity among the trials was very low (*I*^2^ = 4%; *p* = 0.37). Additionally, one non-randomized study (18) reported the occurrence of gastrointestinal bleeding; however, as it was the sole observational study providing this endpoint, it was excluded from the meta-analysis synthesis to maintain methodological consistency between study designs. The single-study estimate from this N-RCT was consistent with the overall findings of the randomized evidence, showing no significant association between the intervention and increased bleeding risk.

Publication bias for gastrointestinal bleeding was assessed using visual inspection of the funnel plot and Egger’s test, both of which indicated no significant evidence of bias (*p* > 0.05; Supplementary Figures S7B,C). “Leave-one-out” sensitivity analysis (Supplementary Figure S7D) further confirmed the robustness of the primary analysis, with the pooled RR remaining stable and its statistical significance unaffected by the exclusion of any single study.

### Other indicators

#### Length of ICU stay

**Figure 8:**
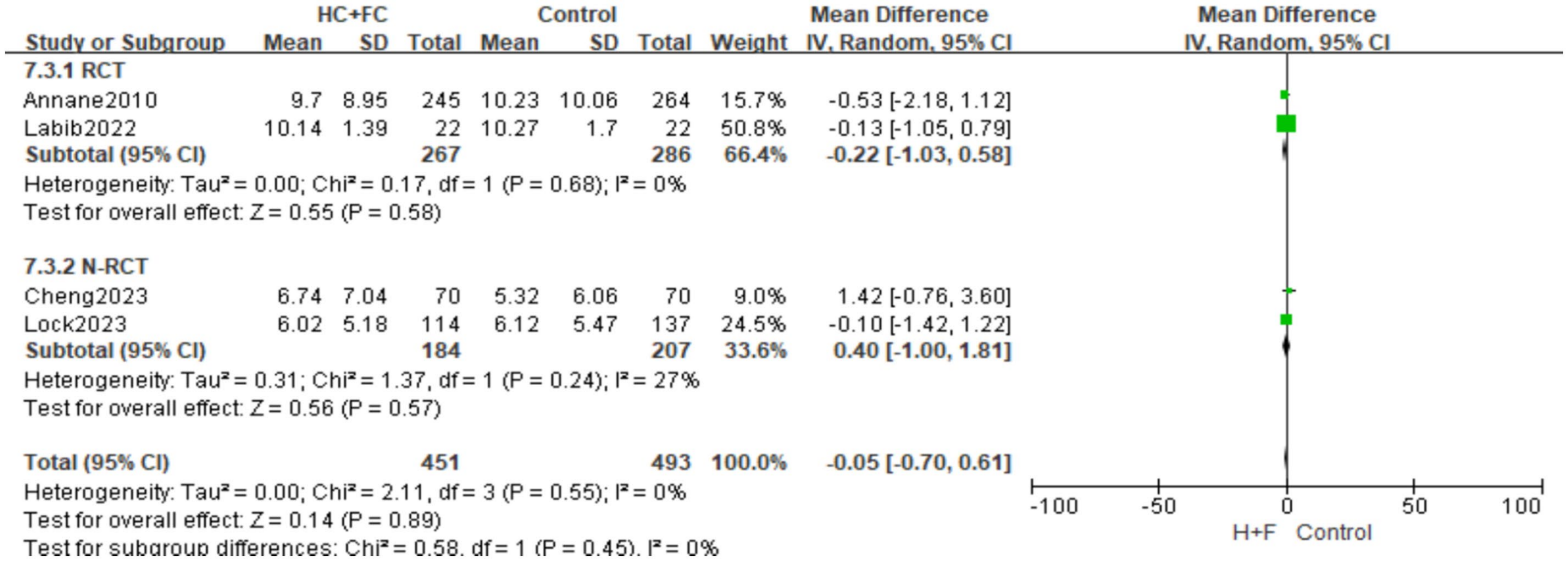
Forest plot of length of stay in ICU.

A total of four studies (18, 21–23) have examined the length of stay in the ICU, involving 944 patients. The pooled analysis of the two RCTs (*n* = 553) showed no significant difference in ICU length of stay between the HC + FC group and the control group [MD: −0.22 days; 95% CI: (−1.03, 0.58); *p* = 0.58], with no observed statistical heterogeneity (*I*^2^ = 0%; *p* = 0.68). Similarly, the meta-analysis of the two N-RCTs (*n* = 391) indicated that the intervention did not significantly affect the duration of ICU stay [MD: 0.40; 95% CI: (−1.00, 1.81); *p* = 0.57, Figure 8], with low heterogeneity (*I*^2^ = 27%; *p* = 0.24). The overall pooled effect across all four studies demonstrated that the combination of hydrocortisone and fludrocortisone did not significantly shorten or prolong the length of ICU stay [MD: −0.05; 95% CI: (−0.70, 0.61); *p* = 0.89]. No significant difference was found between the RCT and N-RCT subgroups (*p* = 0.45; *I*^2^ = 0%, Figure 8), suggesting consistent results across different study designs.

#### Length of hospital stay

**Figure 9:**
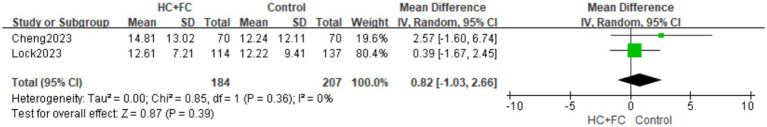
Forest plot of hospital stay time.

Two non-randomized controlled trials (N-RCTs) (18, 23) provided data on the duration of hospital stay. The pooled analysis of these observational studies showed no significant difference in hospital length of stay between patients receiving the combination therapy and the control group [MD: 0.82; 95% CI: (−1.03, 2.66); *p* = 0.39, Figure 9]. No statistical heterogeneity was observed between the included studies (*I*^2^ = 0%; *p* = 0.36). In addition to the observational evidence, one RCT (21) reported on the duration of hospital stay. However, to maintain methodological rigor, this single RCT was not pooled with the N-RCT data. The findings from this individual trial were consistent with the observational evidence, showing no significant impact of HC + FC on total hospital stay duration.

#### Subgroup analysis by comparator type and evidence tier

To further explore the potential survival benefit of hydrocortisone plus fludrocortisone (HC + FC) and address the clinical uncertainty regarding its incremental value over hydrocortisone (HC) alone, we conducted a stratified analysis based on comparator types and study designs (Table 3).

**Table 3:** Subgroup analysis of clinical outcomes stratified by comparator type and study design.

Comparator subgroup	Study design	Title	No. of studies	Total patients	*I* ^2^	RR/MD	95% CI	*p*
HC + FC vs. placebo	RCT	28-day mortality	3 (15, 20, 24)	2,101	5%	0.84	0.76, 0.94	0.002
		90-day mortality	2 (15, 24)	1,803	43%	0.82	0.71, 0.94	0.006
		Hospital mortality	3 (15, 20, 24)	2,101	14%	0.85	0.77, 0.94	0.002
		Reinfection rate	3 (15, 20, 24)	2,101	15%	1.13	1.01, 1.26	0.03
		Gastrointestinal bleeding	3 (15, 20, 24)	2,101	8%	0.86	0.60, 1.22	0.40
HC + FC vs. HC alone	RCT	Reinfection rate	2 (21, 22)	553	17%	1.38	0.86, 2.20	0.19
		Length of stay in the ICU	2 (21, 22)	553	0%	−0.22	−0.13, 0.58	058
	N-RCT	28-day mortality	3 (16, 18, 23)	88,666	15%	0.99	0.91, 1.08	0.79
		90-day mortality	2 (18, 23)	391	0%	0.92	0.77, 1.09	0.34
		Hospital mortality	3 (16, 18, 23)	88,666	48%	0.95	0.80, 1.13	0.60
		Length of stay in the ICU	2 (18, 23)	391	27%	0.40	−1.00, 1.81	0.57
		Length of hospital stay	2 (18, 23)	391	0%	0.82	−1.03, 2.66	0.39

RR, relative risk; MD, mean difference; 95% CI, 95% confidence interval.

HC, hydrocortisone; FC, fludrocortisone; RCT, randomized controlled study; N-RCT, non-randomized controlled study; ICU, intensive care unit.

In the subgroup comparing HC + FC against placebo, the evidence was derived solely from randomized controlled trials (RCTs). The pooled estimates demonstrated that the 28-day mortality rate, 90-day mortality rate, and in-hospital mortality rate of patients in the HC + FC group were lower than those of the placebo group (*p* < 0.05). Heterogeneity remained low across these mortality outcomes (*I*^2^ ranging from 5 to 43%). Notably, while reinfection rates were slightly higher in the HC + FC group (RR 1.13; *p* = 0.03), no significant increase in gastrointestinal bleeding was observed (*p* = 0.40).

When evaluating the incremental benefit of adding fludrocortisone to hydrocortisone, the results were less definitive across different evidence tiers. Data from 2 RCTs (*n* = 553) showed no significant difference in reinfection rates or ICU length of stay (*p* > 0.05), providing limited causal evidence for a distinct advantage of the combination over HC monotherapy. Analysis of retrospective cohorts showed that HC + FC was associated with a neutral effect on 28-day, 90-day, and hospital mortality compared to HC alone (*p* > 0.05). These findings align with the RCT results but should be interpreted as associative, given the potential for residual confounding in observational designs. Both ICU and hospital length of stay showed no significant reduction in either RCT or N-RCT subgroups, suggesting that the combination does not significantly accelerate clinical recovery beyond standard steroid therapy.

## Discussion

This systematic review and meta-analysis provides a comprehensive evaluation of the efficacy and safety of hydrocortisone plus fludrocortisone (HC + FC) in adult patients with septic shock. Our primary findings indicate that compared to placebo, the HC + FC regimen is associated with a significant reduction in 28-day, 90-day, and in-hospital mortality, a conclusion robustly supported by high-quality randomized controlled trials (RCTs). However, a critical nuanced finding emerged regarding the “incremental benefit” of this combination: when compared directly against hydrocortisone (HC) alone, no significant survival advantage or improvement in resource utilization was observed in large-scale observational cohorts. While the combination did not increase the risk of gastrointestinal bleeding, it was associated with a higher rate of reinfection compared to placebo.

Sepsis is a major global public health problem (25). Although the understanding of sepsis continues to improve, the mortality of sepsis remains high. Several studies (26, 27) have estimated that the mortality rate of hospitalized sepsis patients is about 30 to 50%. Septic shock mainly causes microcirculation disturbance, tissue perfusion dysfunction, stimulates inflammatory response, increases the incidence of multiple organ dysfunction syndrome (MODS), and thus increases the mortality of patients (28). Although the pathogenesis of septic shock has not yet been fully clarified, some studies have found that there is immune cell apoptosis in sepsis and septic shock (29, 30). The therapeutic effect of glucocorticoids on severe infections was first proposed in 1951. Since then, glucocorticoids have been widely known to inhibit the inflammatory response in patients with sepsis (31) and have been used for more than 60 years in the management of patients with severe infections. Early studies have shown (32) that the use of large doses of steroid hormones can significantly reduce the mortality of patients with septic shock, which led to their widespread investigation. However, a randomized controlled trial (11) showed that the use of high doses of glucocorticoids increased the chance of superinfection and subsequently increased the mortality of patients with severe sepsis and septic shock. Studies in the 1990s showed that the application of physiological doses of glucocorticoids could improve hemodynamic parameters and reduce mortality (14). Therefore, contemporary clinical practice has shifted towards the use of low-to-moderate doses of glucocorticoids to balance efficacy and safety. Despite a number of clinical studies, the use of glucocorticoids in patients with sepsis is still controversial, and clinical guidance is still unclear.

Hydrocortisone is a glucocorticoid, which has anti-inflammatory, anti-toxic, anti-allergic and anti-shock effects (33). Available data (34) suggest that hydrocortisone increases tissue perfusion and helps prevent ischemic injury of vital tissues and organs. However, the ADRENAL trial (35), which compared the efficacy of a daily intravenous infusion of 200 mg hydrocortisone versus placebo for 7 days in patients with septic shock, showed no significant difference in 90-day mortality between the two groups. With the in-depth study of glucocorticoid treatment in sepsis, it has been discovered that supplementing mineralocorticoids in patients with sepsis may bring clinical benefits. A large clinical study (15) comparing the effects of 200 mg of hydrocortisone and fludrocortisone per day versus placebo in patients with severe septic shock showed a decrease in 90-day mortality in the steroid group, as well as earlier reversal of shock and relief from mechanical ventilation. Septic shock is usually accompanied by dysregulation of the renin-angiotensin-aldosterone system and impaired mineralocorticoid activity. Fludrocortisone is a mineralocorticoid that can enhance the vascular responsiveness to epinephrine, promote sodium and fluid retention, and stabilize the endothelial barrier function, thereby increasing effective circulating blood volume and tissue perfusion (36, 37). Moreover, the activation of mineralocorticoid receptors has been shown to regulate inflammatory signal transduction and endothelial integrity, suggesting that it may have potential in alleviating microcirculation dysfunction in the early stage of septic shock. Consequently, the combination of hydrocortisone and fludrocortisone offers a mechanistically plausible strategy to address both glucocorticoid insufficiency and mineralocorticoid dysfunction in septic shock.

A total of eight articles were included in this study, including five high-quality RCT studies and three retrospective studies. A critical aspect of this meta-analysis is the rigorous differentiation between randomized and observational evidence to prevent inappropriate causal inference. Our risk of bias assessment revealed that the five included RCTs were generally of high methodological quality. However, the three retrospective observational studies inherently carry a higher risk of selection and detection bias. Specifically, as the choice to add fludrocortisone in real-world practice may be influenced by the severity of the patient’s shock or the clinician’s preference, which are factors not always fully captured in retrospective data. The dominance of the Bosch study (16) cohort, warrants specific attention. While its massive sample size provides high statistical power, it also heavily influences the pooled estimates for the N-RCT subgroup. Our stratified analysis shows that despite its weight, the Bosch study showed a statistically neutral association with 28-day mortality, which aligns with our RCT findings when HC + FC was compared against HC alone. To ensure the robustness of our conclusions and mitigate the risk of this single large cohort skewing the results, we employed random-effects models and “leave-one-out” sensitivity analyses. These analyses confirmed that the absence of a significant incremental benefit of fludrocortisone over hydrocortisone alone was consistent across various datasets, reinforcing the stability of our findings. Furthermore, the clinical heterogeneity observed in secondary outcomes, such as the increased reinfection rate compared to placebo, must be contextualized within the varying definitions of “secondary infection” across trials. While the combination therapy addresses both glucocorticoid and mineralocorticoid deficiencies, the cumulative immunosuppressive effect remains a concern (38). Notably, although the increased risk of reinfection compared to placebo is statistically significant, this did not translate into higher late-phase mortality in the RCT subgroup. This suggests that the early hemodynamic benefits of HC + FC may outweigh the infectious risks, provided that patients are closely monitored for secondary complications. Moreover, the mortality rate of patients with septic shock may be affected by persistent immune dysfunction, secondary infections, progressive organ failure, and potential comorbidities, and short-term corticosteroid treatment may not immediately improve these factors (39). In terms of safety, there were no statistically significant differences in the incidence of gastroduodenal bleeding. Compared with placebo, hydrocortisone plus fludrocortisone was associated with a higher overall re-infection rate, whereas no difference was observed relative to hydrocortisone alone, suggesting that this risk is primarily related to glucocorticoid therapy rather than mineralocorticoid supplementation. In addition, it is worth noting that hydrocortisone combined with fludrocortisone did not reduce the length of ICU stay and hospital stay in patients with septic shock. This finding may reflect that the length of ICU stay and total hospital stay are not only influenced by hemodynamic stability but also by the resolution of organ dysfunction, the occurrence of complications, rehabilitation needs and other factors. Hydrocortisone plus fludrocortisone primarily exerts its benefit during the early hemodynamic phase of septic shock, whereas late mortality and recovery-related outcomes are driven by immunoparalysis, secondary complications, and organ repair processes that are less responsive to short-term corticosteroid supplementation. Although combined treatment may help to reverse shock early, it is unlikely to accelerate the recovery of established organ damage, which is the main reason for the prolonged stay of patients in the ICU and hospital. Furthermore, the discharge criteria of different medical institutions vary, and the differences in the baseline disease severity and comorbidities of patients may also further lead to the variability of the hospitalization duration results.

Our findings both align with and extend recent high-quality meta-analyses (40–42) focused on corticosteroids in septic shock. For instance, recent RCT-focused reviews (36) have consistently reported that corticosteroids can accelerate shock reversal, yet their impact on mortality remains a subject of debate depending on the pooled population. By specifically isolating the HC + FC vs. HC alone subgroup, our study adds a crucial layer of evidence: the survival benefit observed in major trials like APROCCHS (15) may be a result of the overall steroid effect rather than the unique addition of fludrocortisone. This distinction is critical for clinicians who may be concerned about the logistical complexities or additional side effects of mineralocorticoid supplementation when hydrocortisone monotherapy is already being administered. Furthermore, our inclusion of a massive observational dataset (*n* > 88,000) provides a “real-world” mirror to the RCT evidence. While recent RCT-only meta-analyses (41, 43) may suggest a survival trend for combination therapy, our stratified data show that in clinical practice settings, the incremental survival gain of fludrocortisone remains statistically neutral. This suggests that the physiological rationale for fludrocortisone may overlap with the inherent mineralocorticoid activity of low-dose hydrocortisone. Consequently, the incremental benefit of dual therapy might be limited to specific patient phenotypes, such as those with profound adrenal exhaustion, a hypothesis that remains to be confirmed by prospective, stratified trials rather than inferred from current observational data.

This study also has some limitations. First, the number of studies included in this paper is small, which may be because additional fludrocortisone has not been extensively studied. In addition, three of the included studies in this meta-analysis were from the same researcher-led study, and the time span is relatively long. Our medical technology and quality of care have improved significantly over the years, and the efficacy of dual corticosteroid therapy should be further evaluated in the course of modern treatment of sepsis. Finally, during the safety evaluation, the types of adverse reactions analyzed were relatively few. It is necessary to have long-term and large-sample adverse reaction reports and monitoring results to confirm the results of this study. We look forward to new research reports for further evaluation.

In conclusion, while HC + FC is associated with a clear survival benefit compared to placebo, its incremental superiority over hydrocortisone alone remains statistically unproven across both randomized trials and large-scale real-world cohorts. This suggests a physiological overlap between the two regimens, potentially rendering routine fludrocortisone supplementation unnecessary for the general septic shock population. However, these findings should be interpreted with caution due to the inherent risk of residual confounding in observational data and the observed increase in reinfection risk. Future head-to-head randomized trials are essential to identify specific patient phenotypes, such as those with refractory vasoplegia, who may derive a true marginal benefit from dual corticosteroid therapy.

## Data Availability

The original contributions presented in the study are included in the article/Supplementary material, further inquiries can be directed to the corresponding author.

## Supplementary material

SUPPLEMENTARY Table 1 Detailed PubMed search strategy.
