# Correction: Hydrocortisone combined with fludrocortisone for treatment of adults with septic shock: an updated meta-analysis and systematic review

**DOI:** 10.3389/fmed.2026.1811616

**Published:** 2026-03-02

**Authors:** Alin Sun, Xiang Gao, Zhipeng Gao, Qinghai Zhang, Wenzheng He

**Affiliations:** 1 Department of Critical Care Medicine, Weifang People's Hospital, Weifang, Shandong, China; 2 Department of Critical Care Medicine, The First Affiliated Hospital of Shandong Second Medical University, Weifang, Shandong, China

**Keywords:** fludrocortisone, hydrocortisone, meta-analysis, mortality, septic shock

Author Xiang Gao was erroneously assigned as equal contributing author.

Author Alin Sun is the lead author and Xiang Gao is the second author; they do not share equal contribution. The symbols indicating equal contribution next to the authors' names have been removed.

The original version of this article has been updated.

