# Supplementary material for: Hydrocortisone combined with fludrocortisone for treatment of adults with septic shock: an updated meta-analysis and systematic review
Source: Front Med (Lausanne). 2026 Feb 11;13:1755626. doi: 10.3389/fmed.2026.1755626 (PMC12932585; doi:10.3389/fmed.2026.1755626)
Supplement: SUPPLEMENTARY Table 1 — Detailed PubMed search strategy. [file Table_1.docx]

## PubMed Search Strategy (Table S1)

**Date of Search:** March 1, 2025 **Database:** PubMed (via National Library of Medicine)

| Step | Search Terms |
| --- | --- |
| #1 (Septic Shock) | "Shock, Septic"[MeSH] OR "Septic Shock" OR "Toxic Shock Syndrome" OR "Endotoxic Shock" OR "Sepsis"[MeSH] |
| #2 (Hydrocortisone) | "Hydrocortisone"[MeSH] OR "Hydrocortisone" OR "Cortisol" OR "Cortics" OR "Compound F" |
| #3 (Fludrocortisone) | "Fludrocortisone"[MeSH] OR "Fludrocortisone" OR "9-alpha-Fluorohydrocortisone" OR "Florinef" |
| #4 (Combination) | "Drug Therapy, Combination"[MeSH] OR "combined" OR "combination" OR "addition" OR "plus" |
| #5 (Final Query) | #1 AND #2 AND #3 AND #4 |
