# Supplementary material for: Hydrocortisone combined with fludrocortisone for treatment of adults with septic shock: an updated meta-analysis and systematic review
Source: Front Med (Lausanne). 2026 Feb 11;13:1755626. doi: 10.3389/fmed.2026.1755626 (PMC12932585; doi:10.3389/fmed.2026.1755626)
Supplement: Supplementary file 2 [file Supplementary_file_1.docx]

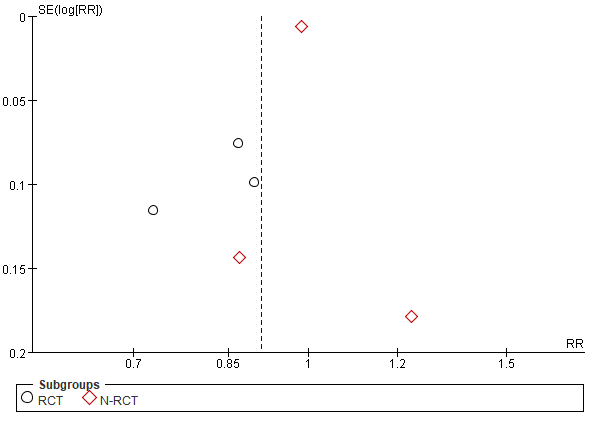


**Figure S3B** Publish bias funnel plots of 28-day mortality rate

**Figure S3C** Begg's funnel plots of 28-day mortality rate

**Figure S3D** Sensitivity analysis of 28-day mortality rate


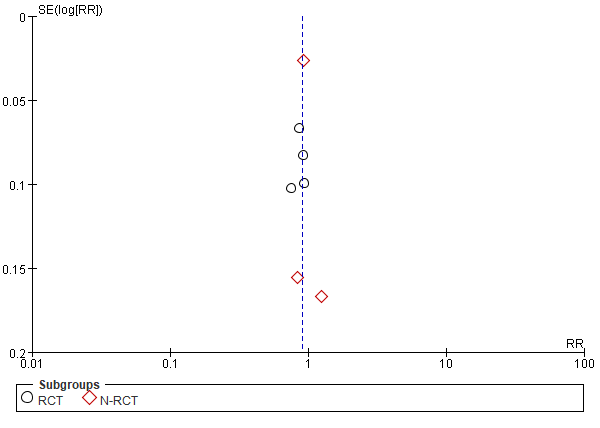


**Figure S4B** Publish bias funnel plots of hospital mortality rate

**Figure S4C** Begg's funnel plots of hospital mortality rate

**Figure S4D** Sensitivity analysis of hospital mortality rate


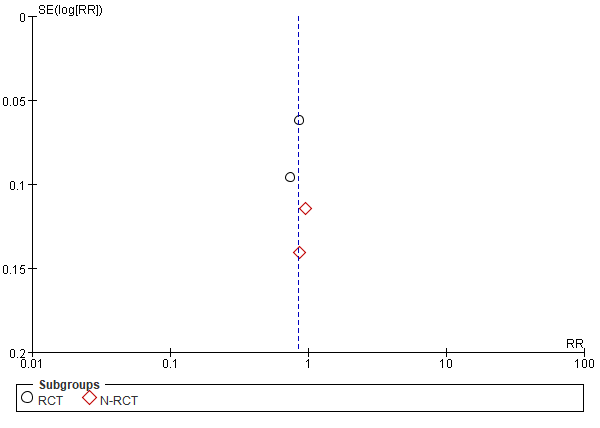


**Figure S5B** Publish bias funnel plots of 90-day mortality rate

**Figure S5C** Begg's funnel plots of 90-day mortality rate

**Figure S5D** Sensitivity analysis of 90-day mortality rate


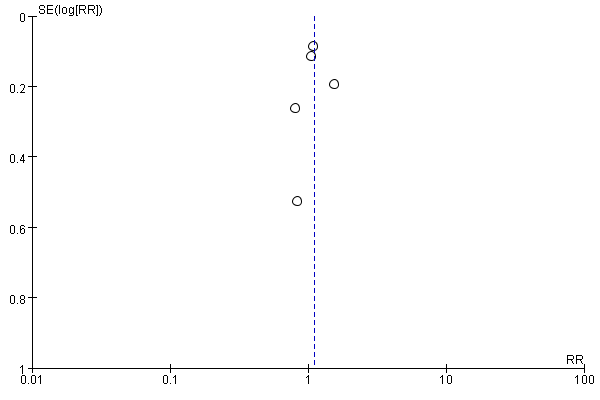


**Figure S6B** Publish bias funnel plots of Re-infection rate

**Figure S6C** Begg's funnel plots of Re-infection rate

**Figure S6D** Sensitivity analysis of Re-infection rate


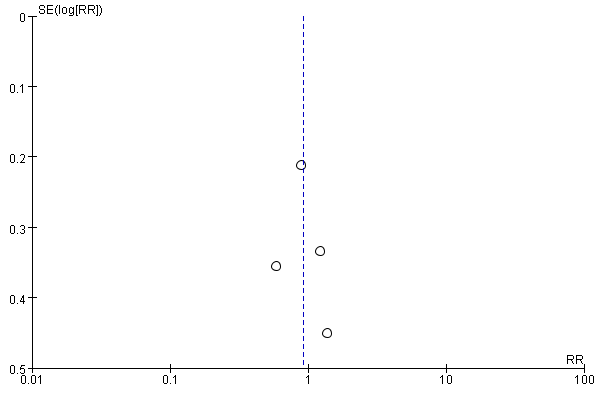


**Figure7B** Publish bias funnel plots of the incidence rate of gastric and duodenal bleeding

**Figure7C** Begg's funnel plots of the incidence rate of gastric and duodenal bleeding

**Figure7D** Sensitivity analysis of the incidence rate of gastric and duodenal bleeding.
